# Norepinephrine protects against cochlear outer hair cell damage and noise-induced hearing loss via α_2A_-adrenergic receptor

**DOI:** 10.1186/s12868-024-00845-4

**Published:** 2024-01-30

**Authors:** Chaoyong Tian, Yang Yang, Renfeng Wang, Yao Li, Fei Sun, Jun Chen, Dingjun Zha

**Affiliations:** Department of Otolaryngology Head and Neck Surgery, Xijing Hospital, Air Force Medical University, Xi’an, Shaanxi 710032 China

**Keywords:** α_2A_- adrenergic receptor, Noise-induced hearing loss, Outer hair cells, Sympathetic nervous system, Superior cervical ganglion

## Abstract

**Background:**

The cochlear sympathetic system plays a key role in auditory function and susceptibility to noise-induced hearing loss (NIHL). The formation of reactive oxygen species (ROS) is a well-documented process in NIHL. In this study, we aimed at investigating the effects of a superior cervical ganglionectomy (SCGx) on NIHL in Sprague-Dawley rats.

**Methods:**

We explored the effects of unilateral and bilateral Superior Cervical Ganglion (SCG) ablation in the eight-ten weeks old Sprague-Dawley rats of both sexes on NIHL. Auditory function was evaluated by auditory brainstem response (ABR) testing and Distortion product otoacoustic emissions (DPOAEs). Outer hair cells (OHCs) counts and the expression of α_2A_-adrenergic receptor (AR) in the rat cochlea using immunofluorescence analysis. Cells culture and treatment, CCK-8 assay, Flow cytometry staining and analysis, and western blotting were to explore the mechanisms of SCG fibers may have a protective role in NIHL.

**Results:**

We found that neither bilateral nor unilateral SCGx protected the cochlea against noise exposure. In HEI-OC1 cells, H_2_O_2_-induced oxidative damage and cell death were inhibited by the application of norepinephrine (NE). NE may prevent ROS-induced oxidative stress in OHCs and NIHL through the α_2A_-AR.

**Conclusion:**

These results demonstrated that sympathetic innervation mildly affected cochlear susceptibility to acoustic trauma by reducing oxidative damage in OHCs through the α_2A_-AR. NE may be a potential therapeutic strategy for NIHL prevention.

**Supplementary Information:**

The online version contains supplementary material available at 10.1186/s12868-024-00845-4.

## Background

Noise-induced hearing loss (NIHL) occurs when the sensitive structures in the inner ear are damaged by loud sound. Exposure to workplace noise for 10 years or more increased the odds of having any kind of hearing loss by a ratio of 2.4 and an increased risk ratio of 6.8 for moderate-to-severe hearing loss [1]. Approximately 22 million workers in the United States (US) are exposed to hazardous noise at work [2]. In China, it’s estimated that approximately 80 million workers exposed to hazardous noise [3].

Following superior cervical ganglionectomy (SCGx) the norepinephrine (NE) tissue levels were decreased after several hours [4]. The sympathetic nervous system (SNS) demonstrated a protective effect against noise-induced damage [5–7]. Presynaptic activation of α_2_-adrenergic receptors (ARs) reduces sympathetic nerve tone by inhibiting the release of catecholamines. A direct activation of postsynaptic receptors on vascular smooth muscle cells causes vasoconstriction, with an increased vascular resistance leading to a decreased blood flow in the cochlea [8]. Cochlear blood flow (CoBF) was reduced during loud sound stimulation [9], with a different response depending on sound level [9–11]. One potential mechanism of NIHL refers to a reduced CoBF during high-level noise exposure [12]. SNS may reduce the blood flow or the permeability of blood vessels of the tympanic lip, impairing the supply of protective factors which the cochlea requires [13, 14]. Secondly, SNS may physiologically activate ion pumps on the hair cell membrane, facilitating the recovery of the internal environment from disturbances caused by noise exposure [15, 16]. Thirdly, SNS may regulate the excitability of afferent and efferent nerves to the cochlea, decreasing the cochlear susceptibility to noise-induced damage [14, 17–19]. As of today, protective mechanisms of SNS for NIHL are not completely clear.

An excess of reactive oxygen species (ROS) is presumably involved in cochlear noise-induced damage [20]. Oxidative stress may contribute to NIHL, impairing sensory hair cells [21]. After noise exposure, ROS may promote cell damage and cell adaptation in the cochlea [22]. In this study, we assessed the impact of a SCGx on NIHL in Sprague-Dawley rats. In addition, we evaluated the H_2_O_2_-induced oxidative damage in HEI-OC1 cells in vitro. NE may prevent ROS-induced oxidative stress in OHCs and NIHL through the α_2A_-AR. In summary, we hypothesized that a SCGx may change susceptibility to NIHL in Sprague-Dawley rats.

## Materials and methods

### Animals

Sprague-Dawley rats were purchased from the Laboratory Animal Center of the Air Force Medical University. All the animals in this study should have a good hearing without hearing loss caused by tympanitis, drug, noise, genetic problem, and so on. All rats had been raised with sufficient food and water in a tranquil animal cage (the plastic box with railing cover) for 5 days prior to the test (the sound levels were 20–30 dB in each cage in which every four rats were housed). We divided twenty-four adult Sprague-Dawley rats of both sexes (eight-ten weeks old, weighing 180–250 g) in three groups (*n* = 8). Bilateral SCGx, unilateral SCGx and sham surgery were performed in the three groups. Rats were housed under standard conditions (temperature 23 ± 2 °C, humidity 60 ± 5%, 12 h light/dark cycle, air cleanliness level 7, and four rats per cage). This study was approved by the Institutional Animal Care and Use Committee of the Air Force Medical University. All animal experiments complied the National Institutes of Health Guide for the Care and Use of Laboratory Animals (NIH publication. 8023, revised in 1978).

### SCGx

**Fig. 1:**
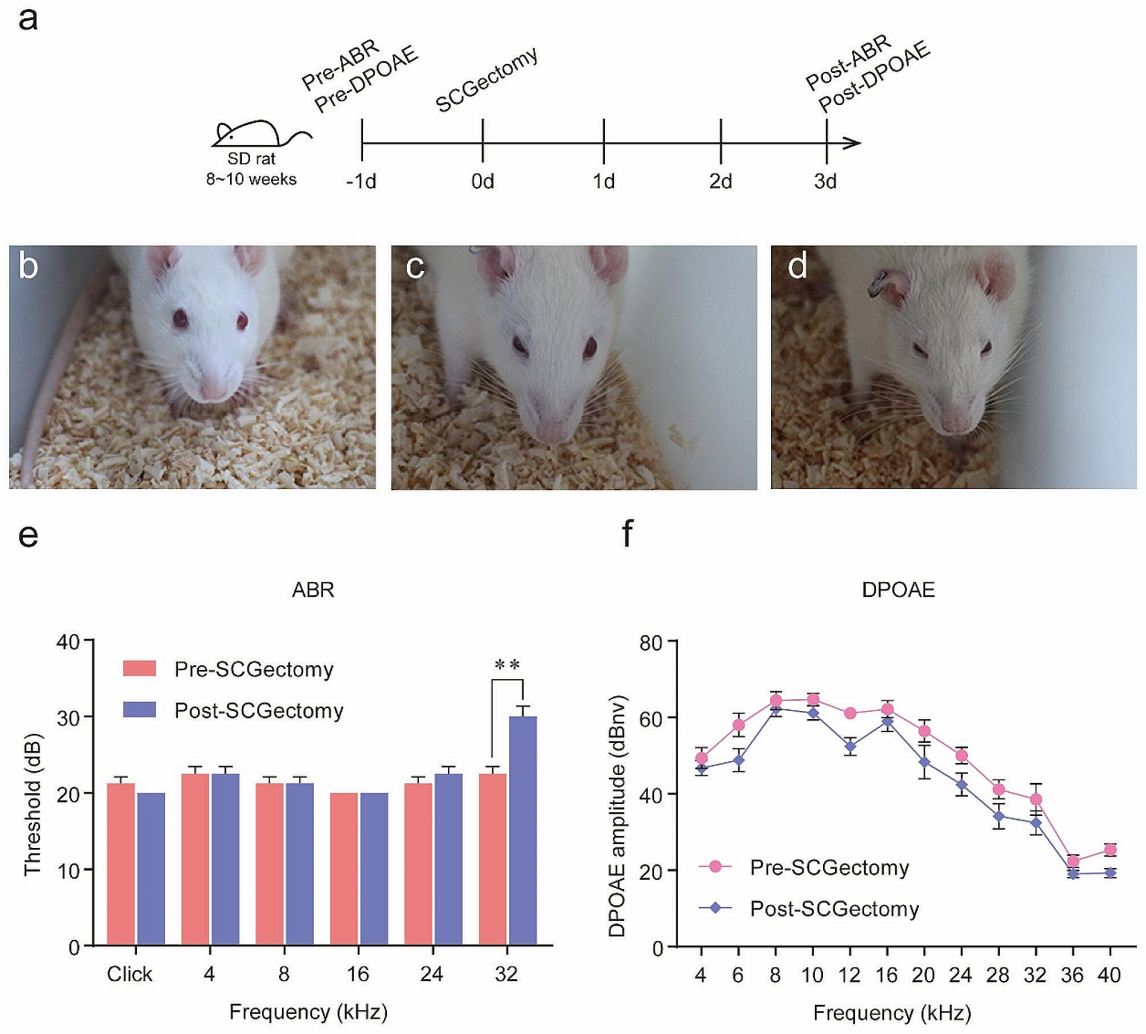
Audiological characterization of pre-SCGx and post-SCGx rats (mean ± standard deviation). **a** In vivo experiment procedures. **b** Horner’s syndrome was not observed after sham operation. **c** Unilateral blepharoptosis was observed after right SCGx. **d** Horner’s syndrome was present after bilateral SCGx. **e** Comparison of ABR thresholds between pre-SCGx and post-SCGx rats. **f** Comparison of DPOAEs amplitude between pre-SCGx and post-SCGx rats. ** *p* < 0.01

The study procedures are briefly described in Fig. 1a. Rats were anesthetized as previously described [23, 24]. Animals were anaesthetized by intraperitoneal injection of a mixture of ketamine (57.14 mg/kg) and xylazine (5.71 mg/kg). In SCGx, the operating procedure as previously described [25]. superior cervical ganglions (SCGs) located behind the carotid bifurcations were removed, either unilaterally or bilaterally. The sham operation employed the same approach except for SCGs resection. Horner’s syndrome appearing on the operated side(s) indicated that SCGx was successful (Fig. 1b-d) [26], animals without bilateral palpebral ptosis and, therefore, with incomplete surgery, could be easily identified and discarded.

### Noise exposure

After a two-week recovery period from surgery, rats were exposed to an octave band of noise (4 kHz) at 115 dB SPL for six continuous hours per day during two consecutive days. The sound exposure chamber was previously described [27]. A Radio Shack Super Tweeter was located above the cages, generated a noise that was then amplified by a power amplifier and delivered to a loudspeaker. Noise levels were confirmed with a sound level meter (Bruel and Kjaer, China) at multiple locations within the cabinet to ensure uniformity of the sound field and were measured before and after exposure to ensure stability.

### Auditory brainstem responses (ABRs)

We measured ABR thresholds in bilateral SCGx, unilateral SCGx and sham groups at 1 h, 3 days, 7 days and 14 days after noise exposure, and the bilateral audition of each rat were examined. In auditory function statistical analysis, the left ear of bilateral SCGx group (*n* = 8 ears) was selected as the test ear, the left ear of unilateral SCGx group (unilateral-ipsilateral SCGx group) (*n* = 8 ears) was selected as the test ear, the right ear of unilateral SCGx group (unilateral-contralateral SCGx group) (*n* = 8 ears) was selected as the test ear, and the left ear of sham surgery group (*n* = 8 ears) was selected as the test ear. After rats were anesthetized, an active electrode was inserted under the skin at the vertex, a reference electrode was inserted in the mastoid area of the test ear and a ground electrode was placed below the root of the tail. The TDT System-III hardware and SigGenRZ/BioSigRZ software (Tucker-Davis Technologies, Alachua, FL, US) were used to collect data. Alternating click stimuli were used for response detection and delivered using a headphone MF11172, which filtered the acoustic range to 100–3000 Hz. The speaker was in this experiment 1 cm away from the outer ear canal of the rat. Stimulus began with 90 dB intensity (21/s) of tone-burst (frequencies of 4, 8, 16, 24 and 32 kHz), with 10 dB decreased once, and then in 5 dB intervals near threshold until no responses were detected. The first wave was used to determine thresholds for each frequency.

### Distortion product otoacoustic emissions (DPOAEs)

The DPOAEs were measured using a TDT WS-8 computer workstation (Tucker-Davis Technologies, Alachua, FL, US). DP-grams of DPOAE amplitudes as a function of f1 and f2 frequencies were represented. Frequencies were acquired with an f2/f1 ratio of 1.2. At 1 h, 3 days, 7 days and 14 days after noise exposure, frequency was incremented from 4 to 40 kHz through 2 and 4 kHz steps. The detection threshold was determined as a DPOAE of 3 dB above the noise floor.

### Immunofluorescence

For immunohistochemistry of OHCs, the cochlea was removed and fixed in 4% paraformaldehyde for 24 h and then decalcified in 10% EDTA solution for 3 to 7 days. After the cochlear tissue was softened, the basement membrane was peeled off under a microscope, and immunofluorescence staining was performed. Cochlear hair cells were labeled with phalloidin (Cytoskeleton, Inc., Denver, CO, USA; PHDR1, 1:200).

For immunohistochemistry of cochlear cryosections, staining procedures were performed as described previously [28]. The cochlear sections were incubated for 40 min at 37°C with a blocking solution made of PBS (0.1% Triton X-100 in PBS) with 5% normal donkey serum (NDS; Jackson ImmunoResearch, USA). The primary antibody was applied to the cochlea sections overnight at 4°C. After rinsing three times in PBS, secondary antibody incubation was performed at room temperature for 2 h. After washing in PBS, HCs were labeled with rhodamine phalloidin (Cytoskeleton, Inc., Denver, CO, USA; PHDR1, 1:200) for 10 minutes at room temperature in the dark. Sections were counterstained with 4’, 6-diamidino-2-phenylindole (DAPI; Solarbio Corporation, Beijing, China, 1:1000) or Hoechst 33,342 (Yeasen, Shanghai, China, 10 µg/mL) for 10 min at room temperature in the dark and mounted on glass slides with Mowiol 4–88 (81381-50G, Sigma Aldrich, Germany) mounting medium.

For primary antibodies, we used α_2A_-AR (ab85570, abcam, 1:200), Dopamine Beta-Hydroxylase (DBH) (DF7060, Affinity Biosciences, 1:500), and β3 (TUJ-1; MAB1637, Millipore, 1:1000). For secondary antibodies, we used Alexa 488 conjugated-donkey anti-rabbit secondary antibody ( A-21,206, Thermo Fisher, 1:200) and Alexa 594 conjugated-goat anti-mouse secondary antibody (A11005, Thermo Fisher, 1:200).

The specimens were observed using a fluorescence microscope (Olympus Corp., Tokyo, Japan). The images were captured using Olympus confocal software FV10-ASW 1.7a and adjusted for brightness and contrast.

### OHCs counts

After auditory functional measurements, we histologically analyzed the influence of SCGx on acoustic trauma. OHCs loss was examined under a fluorescence microscope (40× magnification; Olympus Corp., Tokyo, Japan) from the apex to the base of the cochlea. The percentage of cells lost along the entire basilar membrane was calculated.

### Cells culture and treatment

HEI-OC1, an inner ear cell line, was kindly provided by Dr. Renjie Chai at Southeast University. HEI-OC1 cells were cultured as previously described [29]. The cells were cultured in DMEM (C11995500BT, Gibco, Grand Island, NY, United States) containing 10% FBS (10,099,141, Gibco, Grand Island, NY, United States) and 1% penicillin (SV30010, HyClone, South Logan, UT, United States) in acceptable conditions (5% CO_2_, 37 °C). The cells were subcultured at 80% confluence using 0.25% trypsin/EDTA (25,200,056, Gibco, Grand Island, NY, United States). When cells were cultured to a suitable density, the serum was removed, and cells were washed with PBS three times. We performed in vitro oxidative stress experiments in HEI-OC1 cells to uncover potential NE-mediated mechanisms against NIHL. HEI-OC1 cells are widely used for studying hair cell pathology [20, 30, 31]. For oxidative stress experiment, the cells were subcultured with fresh medium containing H_2_O_2_ at different concentrations (0, 0.2, 0.4, and 0.8 mmol/l) for 1 h, 3 and 5 h. To reduce ROS production, we treated HEI-OC1 cells with NE, a postganglionic sympathetic neurotransmitter. The cells were treated with it at various concentrations (0, 0.1, 1, 5, 10 and 50 µM) with H_2_O_2_ (0, 0.2, 0.4 and 0.8 mmol/l) for 1 h, 3 and 5 h.

### CCK-8 assay

Cultured HEI-OC1 cells were plated in each well of a 96-well cluster dish. After attachment, cells were treated with H_2_O_2_ (0, 0.2, 0.4 and 0.8 mmol/l) for 1 h, 3 and 5 h. Following treatment with NE, cells were incubated for 5 h with the Cell Counting Kit-8 (CCK-8) reagent (100 µl/ml, MCE, Shanghai, China) at 37 °C for 5 h. Finally, absorbance was measured at 450 nm through the microplate reader (Bio-Rad, Hercules, CA, US).

### Flow cytometry staining and analysis

HEI-OC1 cells were treated with or without H_2_O_2_ and NE at 37 °C. Then, the detailed procedures of flow cytometry analysis are described as previously [32]. For the measurement of apoptosis, discarded the supernatants and the cells were resuspended in 1 × annexin-binding buffer. The apoptosis rate was detected by staining with Annexin V-FITC and propidium iodide (Millipore, APOAF-50TST). Finally, all samples were detected on FC500 flow cytometry (Beckman).

### Western blot analysis

Proteins (25 µg) were isolated from cochlear tissues and separated by electrophoresis on universal SDS-PAGE gels. After electrophoresis, protein samples were transferred into a polyvinylidene fluoride membrane (IPVH00010, Millipore, Burlington, MA, USA) and blocked for 1 h with 5% dry milk in tris buffered saline (TBS) and 0.1% tween-20 (TBST). The blots were cut prior to hybridisation with antibodies during blotting, the membranes were incubated with specific primary antibodies: anti-α_1A_-specific polyclonal antibody (1:1000, 19777-1-AP, Proteintech), anti-α_1B_ antibody (1:1000, DF8798, Affinity), anti-α_1D_ antibody (0.2 µg/ml, ab166925, Abcam), anti-α_2A_ polyclonal antibody (1:1000, 14266-1-AP, Proteintech), anti-α_2B_ antibody (1:1000, A8535, ABclonal), anti-α_2C_ antibody (1:1000, DF3108, Affinity), anti-β_1_ antibody (1:1000, bs-0498R, Bioss), anti-β_2_ antibody (1:1000, DF3512, Affinity), anti-β_3_ antibody (1:1000, bs-1063R, Bioss) and anti-GAPDH polyclonal antibody (1:10000, 10494-1-AP, Proteintech) overnight at 4 °C. Then, the membranes were washed in TBST and incubated with secondary antibodies (1:5000 dilution, CWBIO, China) for 1 h followed by chemiluminescent detection (Merck Millipore). The immunoreactive bands were visualized by eBlot Touch Imager (e-Blot, China).

### Statistical analysis

All data are presented as mean ± standard deviation (SD). Data were analyzed with GraphPad Prism software (version 8.3.0, San Diego, CA, US). A two-way analysis of variance (ANOVA) followed by Tukey post-hoc test or independent *t*-test were performed for comparisons. A two-tailed statistical significance was defined as *p* < 0.05.

## Results

### Auditory function before and after SCGx

The thresholds at click, 4, 8, 16, 24 and 32 kHz of all SCGectomized rats were compared before and after SCGx (Fig. 1e). The ABR threshold at 32 kHz was significantly lower in pre-SCGx rats (*n* = 8, two-way ANOVA, *p* < 0.005), whereas no difference was found at click, 4, 8, 16 and 24 kHz between pre-SCGx and post-SCGx rats (*n* = 8, *p >* 0.05).

We further investigated the effects of SCGx on DPOAEs by comparing the thresholds at 4, 6, 8, 10, 12, 16, 20, 24, 28, 32, 36 and 40 kHz before and after SCGx (Fig. 1f). No significant differences in DPOAE response amplitudes were observed at 4, 6, 8, 10, 12, 16, 20, 24, 28, 32, 36 and 40 kHz between pre-SCGx and post-SCGx rats (*n* = 8, *p >* 0.05).

### Effects of SCGx on noise exposure

**Fig. 2:**
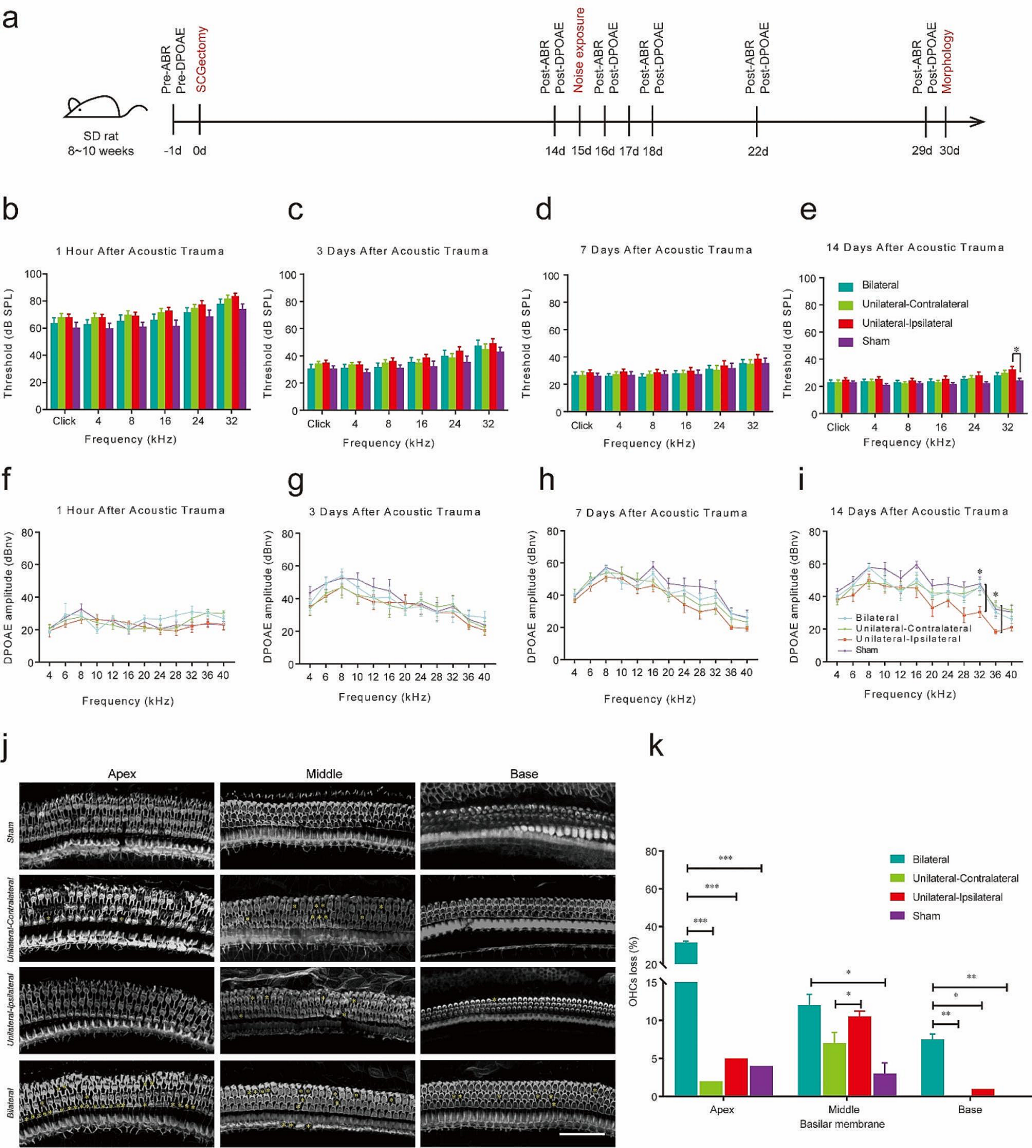
Thresholds of ABR and DPOAEs (mean ± standard deviation) and representative images of the rat cochlea (magnification ×20) showed OHCs loss at 14 days post-noise. **a** In vivo experiment procedures. **b and f** At 1 h post-noise. **c and g** At 3 days post-noise. **d and h** At 7 days post-noise. **e and i** At 14 days post-noise. Data were analysed using a two-way analysis of variance (ANOVA) followed by Tukey post-hoc test (*n* = 8 per group). * *p* < 0.05. **j** Phalloidin labelling showed OHCs survival in the cochlea after SCGx and noise. **k** Quantification of phalloidin-positive OHCs. Data were analysed with Dunnett’s multiple comparisons (repeated measures). Scale bar = 50 μm. ^*^p < 0.05, ^**^p < 0.01, ^***^p < 0.001

To examine the effects of the SCG fibers on noise exposure, we performed the SCGectomy surgical, auditory functions test, and morphological observation (Fig. 2a). At 1 h, 3 days and 7 days after noise exposure, the average threshold was slightly higher in bilateral SCGx, unilateral-contralateral SCGx and unilateral-ipsilateral SCGx groups than in the sham group (Fig. 2b-d). However, the thresholds were indistinguishable among groups for all frequencies tested (*n* = 8, two-way ANOVA, *p* > 0.05). At 14 days after noise exposure, the average threshold was significantly higher in the unilateral-ipsilateral SCGx group at 32 kHz than the sham group (*n* = 8, *p* < 0.01), but the ABR threshold was indistinguishable among groups for the other frequencies tested (*n* = 8, *p* > 0.05) (Fig. 2e).

Figure 2f-i shows the mean DPOAEs at 1 h, 3 days, 7 days and 14 days after noise exposure. At 1 h post-noise exposure, mean DPOAE amplitudes were not significantly different among bilateral SCGx, unilateral SCGx and sham groups at all tested frequencies (*n* = 8, two-way ANOVA, *p* > 0.05) (Fig. 2f). At 3 days and 7 days post-noise exposure, there was no difference among groups (*n* = 8, *p* > 0.05) (Fig. 2g-h). At 14 days post-noise exposure, mean DPOAE amplitudes of the unilateral-ipsilateral SCGx group were lower than amplitudes of the sham group at 32 kHz (*n* = 8, *p* < 0.05), and mean DPOAE amplitudes of the unilateral- ipsilateral SCGx group were lower than amplitudes in the unilateral- contralateral SCGx group at 36 kHz (*n* = 8, *p* < 0.05) (Fig. 2i), while the mean DPOAEs was indistinguishable among groups for the other frequencies tested (*n* = 8, *p* > 0.05).

### The distribution of SCG fibers in inner ear attenuates noise-induced loss of outer hair cells in rats

We explored the OHCs loss in rats exposed to SCGx and noise (Fig. 2j). In the cochlear apex and base, the loss of OHCs in the bilateral SCGx + noise group was higher than the other three groups (*p* < 0.05). In the cochlear middle turn, the OHCs loss in the unilateral-ipsilateral SCGx + noise group was higher compared with the unilateral-contralateral SCGx + noise group (*p* < 0.05), while the OHCs loss in the bilateral SCGx + noise group was higher respect to the sham + noise group (*p* < 0.05). Figure 2k shows the quantifications related to Fig. 2j.

### NE protected against H_2_O_2_-induced oxidative stress in HEI-OC1 cells

**Fig. 3:**
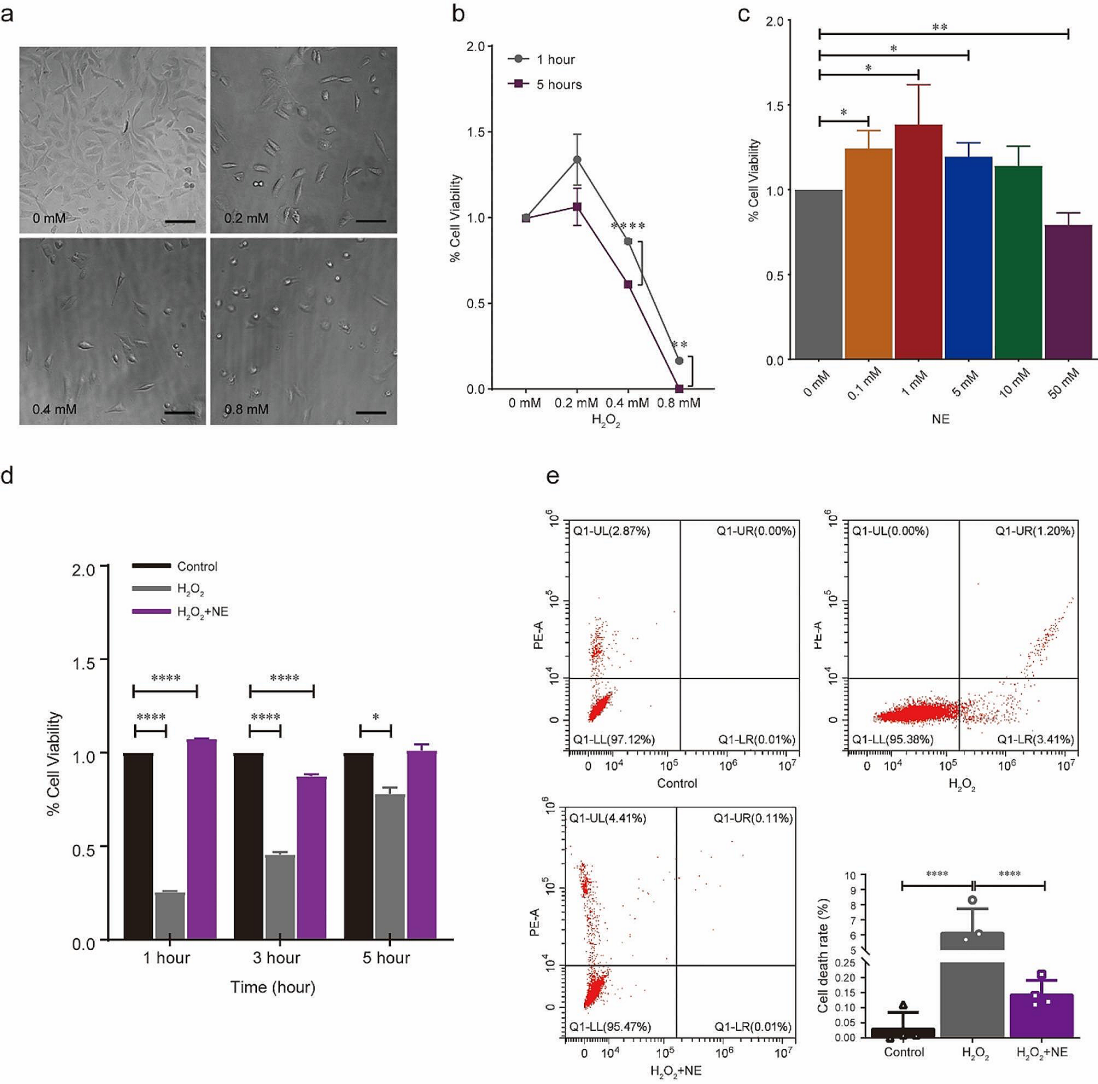
HEI-OC1 cell survival after H_2_O_2_ exposure and NE treatment. **a** Cell images were captured by holographic microscopy. The morphological changes are displayed over time after treatment with H_2_O_2_. Scale bar = 100 mm, *n* = 3. **b** Viability of HEI-OC1 cells at 1 and 5 h after stimulation with 0, 0.2, 0.4 and 0.8 mM of H_2_O_2_. **c** Viability of HEI-OC1 cells at 5 h after stimulation with 0, 0.1, 1, 5, 10 and 50 mM of NE. **d** Effects of NE on HEI-OC1 cell survival after H_2_O_2_ exposure. **e** Quantification of cell apoptosis for different treatments. ^*^p < 0.05, ^**^p < 0.001, ^****^p < 0.0001

As illustrated in Fig. 3a, H_2_O_2_ exposure at 0, 0.2, 0.4 and 0.8 mM caused a dose-dependent reduction in cell viability. When compared with the control group, cell viability declined markedly after 5 h of H_2_O_2_ exposure at 0.2, 0.4 and 0.8 mM. Figure 3b shows the quantifications related to Fig. 3a.

As illustrated in Fig. 3c, the CCK-8 assay was used to evaluate the cytotoxicity of NE on HEI-OC1 cells. When compared with the control group, the cell viability declined in a dose-dependent way after 5 h of NE exposure.

To determine whether NE plays a protective role in oxidative stress injury, we treated HEI-OC1 cells with H_2_O_2_ (0.4 mM) and NE (10 mM) for different points in time. Cell variability were determined using CCK-8 method at 1 h, 3 and 5 h, and a proliferation bar was drawn (Fig. 3d). We found that the cell viability of NE + H_2_O_2_ group was higher than that of the H_2_O_2_ group. Meanwhile, cellular apoptosis was examined using a flow cytometer at 5 h. The results indicated that the percentage of apoptotic and necrotic cells in the NE + H_2_O_2_ group was significantly lower than that in the H_2_O_2_ group (Fig. 3e).

### α_2A_-AR mediated protective effects against NIHL

**Fig. 4:**
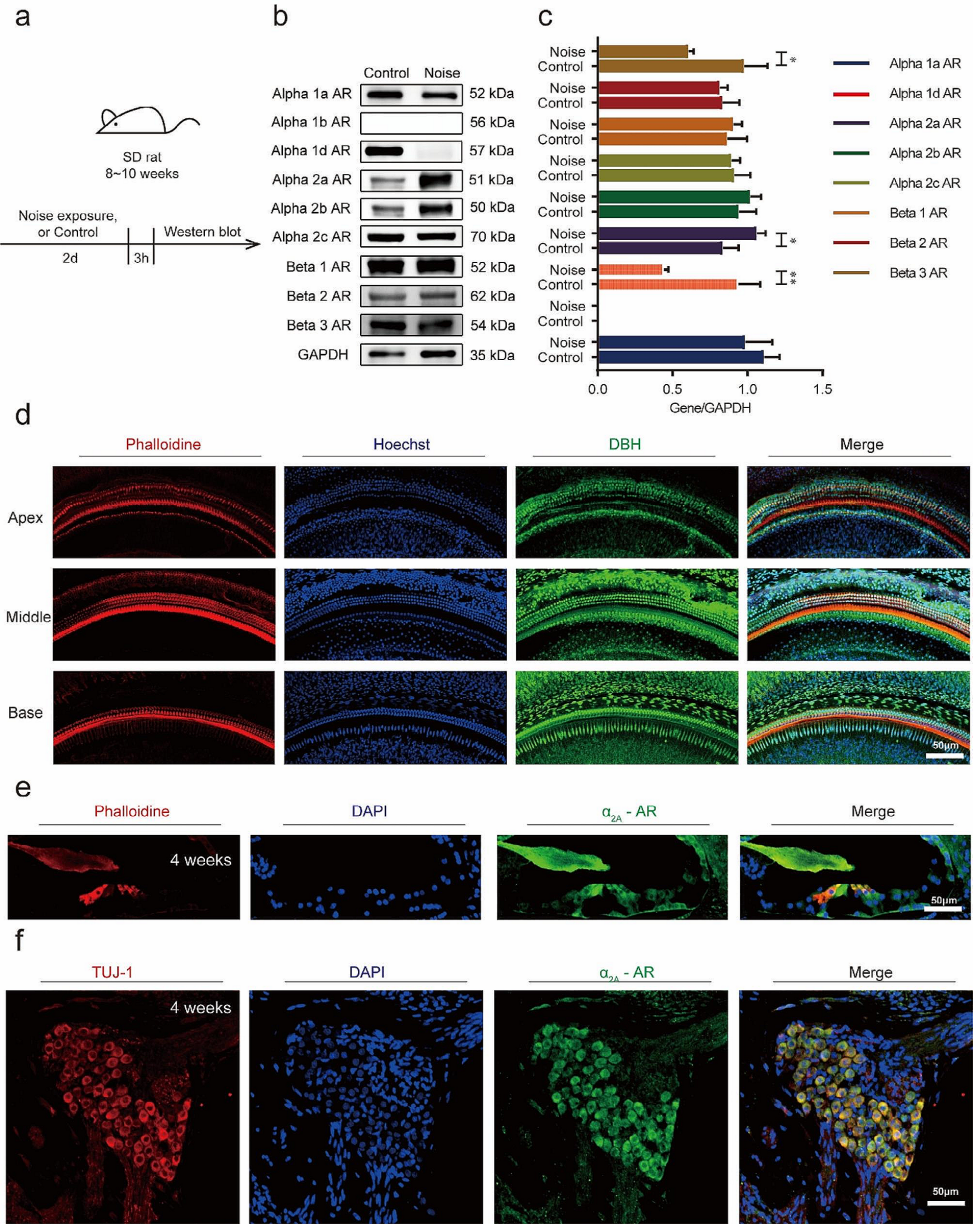
Expression changes of ARs in rats exposed to noise and controls. **a** A schematic diagram illustrating the experimental procedure. **b** Western blot showing the expression changes of ARs in rats exposed to noise and controls (*n* = 6). **c** Quantification of the western blotting results. ^*^p < 0.05, ^**^p < 0.01. **d** Representative images showing DBH immunostaining at different positions along the base-to-apex axis. Rows and columns correspond to different positions along the cochlear axis, respectively, as indicated. Scale bar: 50 μm. **e-f** Representative images showing α_2A_-AR immunostaining at Organ of Corti and SGNs. Scale bar: 50 μm

Western blotting was used to investigate the expression of ARs (Fig. 4a-c). We found that the expression of α_2A_-AR significantly increased in rats exposed to noise compared with controls. Moreover, the expression of α_1D_-AR and β_3_-AR decreased in rats exposed to noise compared with controls (Fig. 4c). DBH, an enzyme that converts dopamine (DA) to NE, to investigate the distribution patterns of DBH and α_2A_-AR in the rat cochlea, we carried out immunofluorescence staining in adult rats. The results showed that DBH were predominantly immunoreactive in the HCs of rat cochlea (Fig. 4d). And α_2A_-AR was expressed in the HCs (Fig. 4e) and the spiral ganglion neurons (SGNs) of rat cochlea (Fig. 4f).

## Discussion

This paper we have three novel findings: [1] Neither bilateral nor unilateral SCGx protected the cochlea against noise exposure [2]. In HEI-OC1 cells, H_2_O_2_-induced oxidative damage and cell death were inhibited by the application of NE [3]. NE may prevent ROS-induced oxidative stress in OHCs and NIHL through the α_2A_-AR.

### Sympathetic innervation and auditory function

CoBF is mainly regulated by sympathetic nerves [17, 33–36]. In central auditory mechanisms, the roles of locus coeruleus (LC) and NE are predominantly inhibitory [37–41]. In post-SCGx rats, DPOAEs exhibited a lower amplitude compared with that observed in pre-SCGx rats. However, the difference was not statistically significant. Our results are consistent with a previous study reporting no changes of DPOAEs after SCGx [7]. Moreover, we showed that pre-noise exposure ABR thresholds at 32 kHz were higher in post-SCGx rats than in pre-SCGx rats, strengthening our DPOAE results.

### Sympathetic innervation and NIHL

The effects of SCGx on auditory function suggested that SCG fibers may have a protective role in NIHL. Previously, SCGx resulted in a reduced susceptibility to NIHL [6, 7, 42–44]. A study conducted in *Dbh*^*−/−*^ mutant mice, which were no more susceptible to damage caused by noise exposure than controls [45]. Another study observed a protective influence of the ipsilateral cervical SNS during electrical stimulation [5]. Protection from temporary threshold shift (TTS) under sedation/anesthesia might be due to a diminished sympathetic influence [46]. After noise exposure, dexmedetomidine displayed protective effects against NIHL [47]. In mice, manipulation of adrenergic inputs to the cochlea confirmed that SNS regulates cochlear blood flow in response to intense loud sound exposure [48]. However, these findings are contradictory. Hence, the objective of this study was to clarify the potential influence of SCGx on noise-induced cochlear injury. The auditory function was assessed via ABRs and DPOAEs. ABRs reflect the auditory nerve activity while DPOAEs are used to assess the function of OHCs [49–51]. In accordance with morphological data, we showed that the auditory function of the unilateral- ipsilateral SCGx group was worse than that in the sham group at 32 kHz and the unilateral- contralateral SCGx group at 36 kHz after noise exposure.

### Possible mechanisms underlying the SNS protective effect on NIHL

We showed that sympathetic innervation affected cochlear susceptibility to acoustic trauma. There are several potential mechanisms explaining how sympathetic fibers could influence noise-induced changes of cochlear function. Upon noise exposure, SNS caused cochlear vasoconstriction, reducing CoBF and aggravating acoustic trauma [43]. Two regulatory systems may be involved, including lateral wall pericyte signaling linking sound-induced metabolic demand to blood flow and a sympathetically activated arteriolar feedback loop influencing CoBF. Sympathetic projections from the stellate ganglion confirmed the functional role of SNS in CoBF regulation [34], suggesting that such regulation might be prevalent when the SCG is removed. In terms of vessel-independent components, cochlear sympathetic fibers previously exacerbated acoustic damage by an unknown mechanism [6]. Unilateral SCGx might alter the overall influence of the olivocochlear efferent system. However, this hypothesis is not currently supported by evidence [6, 43]. The medial and lateral olivocochlear systems are known to protect the cochlea against acoustic trauma. Electrophysiological experiments suggested a direct input of NE-containing neurons to medial olivocochlear neurons [40], revealing that sympathetic fibers may be protective in noise-induced damage by regulating the function of the olivocochlear system. In addition, noradrenergic inputs mediated the function of auditory efferents [52]. Regardless of the precise underlying mechanism, our findings deserve further investigation.

We showed that NE decreased the intracellular ROS levels and increased the HEI-OC1 cells survival. Western blotting analysis detected nine subtypes of ARs in the cochlea of noise-exposed rats and controls. We found that α_2A_-AR were overexpressed after noise exposure, whereas the expression of α_1D_-AR and β_3_-AR was decreased. The sympatholytic effects in the central nervous system and sympathetic ganglia are mediated through the α_2A_-AR. Activation of α_2A_-AR causes a decreased release of catecholamines, resulting in vasodilation [53]. The α_2A_-AR are G protein-coupled receptors (GPCR) which can inhibit adenylyl cyclase and the consequent accumulation of intracellular cAMP [54, 55]. These findings suggested that α_2A_-AR may exert a protective effect against OHC damage and NIHL. Further studies on α_2A_-AR are needed to better understand the involved signaling pathways. Our study provides a theoretical basis for understanding the role of the adrenergic system and its therapeutic potential against NIHL.

## Conclusions

**Fig. 5:**
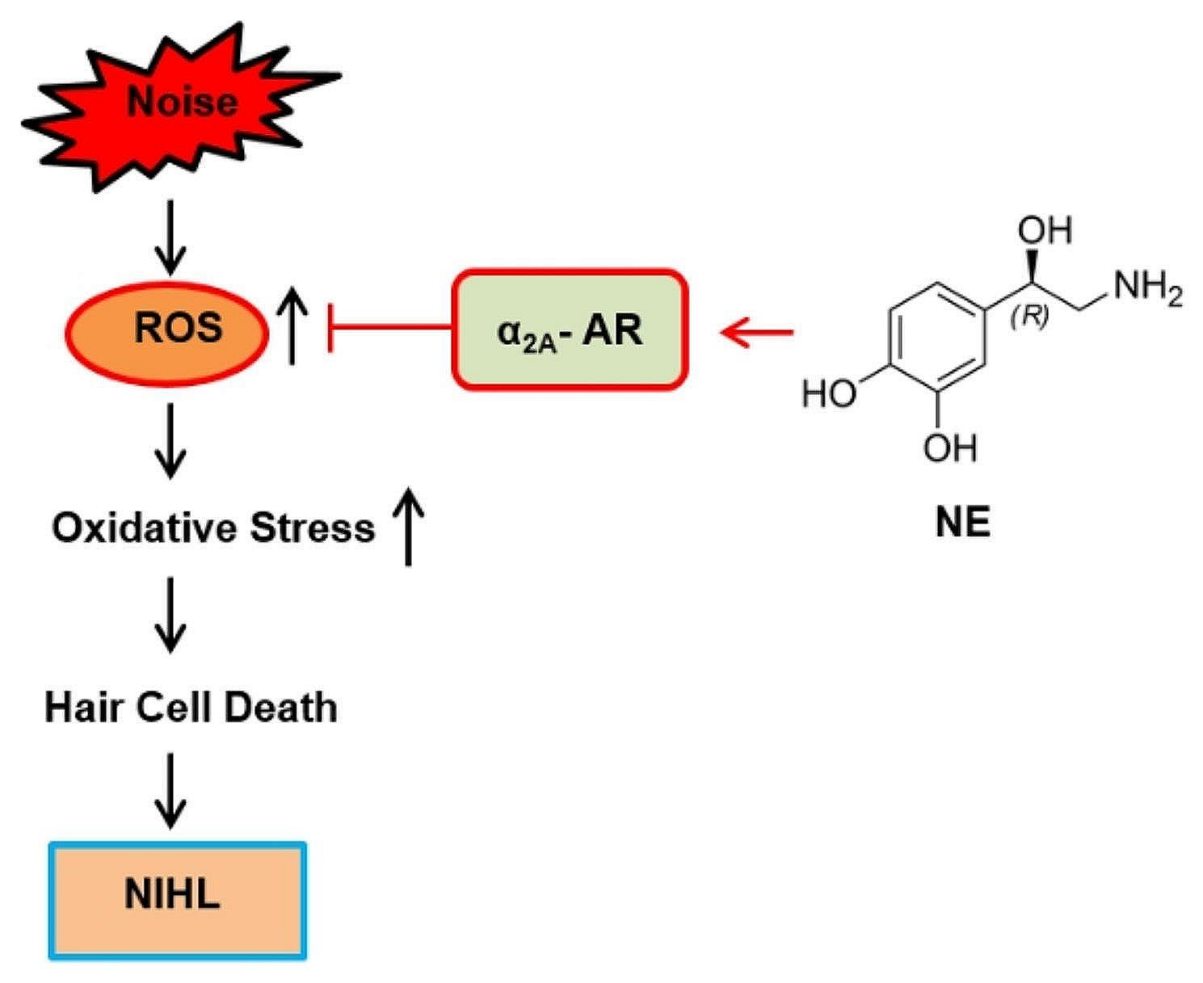
Schematic diagram of potential protective mechanisms of NE against NIHL via α_2A_-AR. The levels of ROS increased after noise exposure in OHCs, leading to cell death and NIHL. Treatment with NE, an activator of α_2A_-AR, attenuated oxidative stress and protected from cell injury

We showed that sympathetic fibers do exert some mild effects on auditory function, however, the underlying mechanism requires further investigation. Neither bilateral nor unilateral SCGx exerted a protective effect on cochlear damage induced by noise. NE may prevent ROS-induced oxidative stress in OHCs through the α_2A_-AR (Fig. 5). Our findings open a new perspective in the treatment of NIHL.

### Electronic supplementary material

Below is the link to the electronic supplementary material.

Supplementary Material 1

## Data Availability

All data generated or analysed during this study are included in this published article. The datasets used and/or analysed during the current study are available from the corresponding author on reasonable request.

## Abbreviations

ABR: Auditory brainstem response
ANOVA: Analysis of variance
CoBF: Cochlear blood flow
DPOAEs: Distortion product otoacoustic emissions
DA: Dopamine
GPCR: G protein-coupled receptors
I/O: Input/output
NE: Norepinephrine
NIHL: Noise-induced hearing loss
OHC: Outer hair cell
SCG: Superior cervical ganglion
SCGx: Superior cervical ganglionectomy
SNS: Sympathetic nervous system
