# Supplementary material for: Norepinephrine protects against cochlear outer hair cell damage and noise-induced hearing loss via α2A-adrenergic receptor
Source: BMC Neurosci. 2024 Jan 30;25:5. doi: 10.1186/s12868-024-00845-4 (PMC10829207; doi:10.1186/s12868-024-00845-4)

Fig.S1. The gel images for Fig.4.

1. Alpha 1a AR (19777-1-AP)

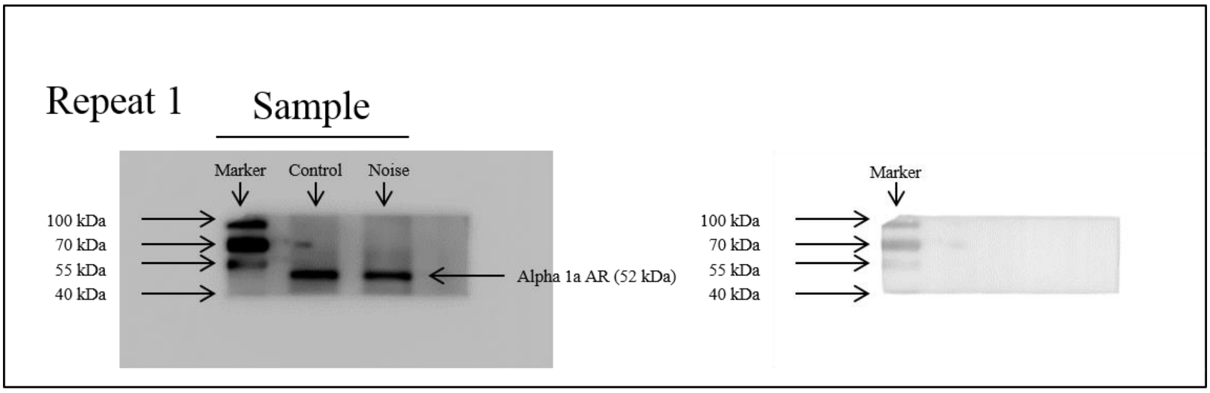

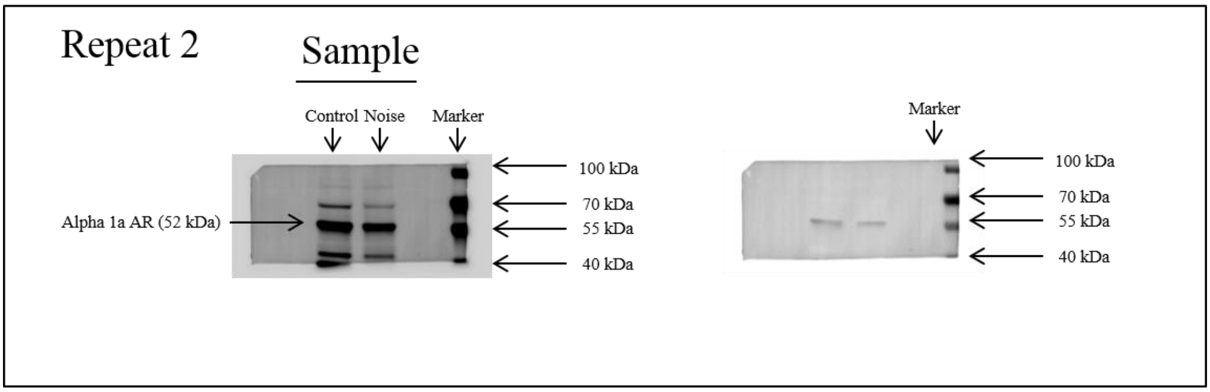

2. Alpha 1b AR (DF8798)

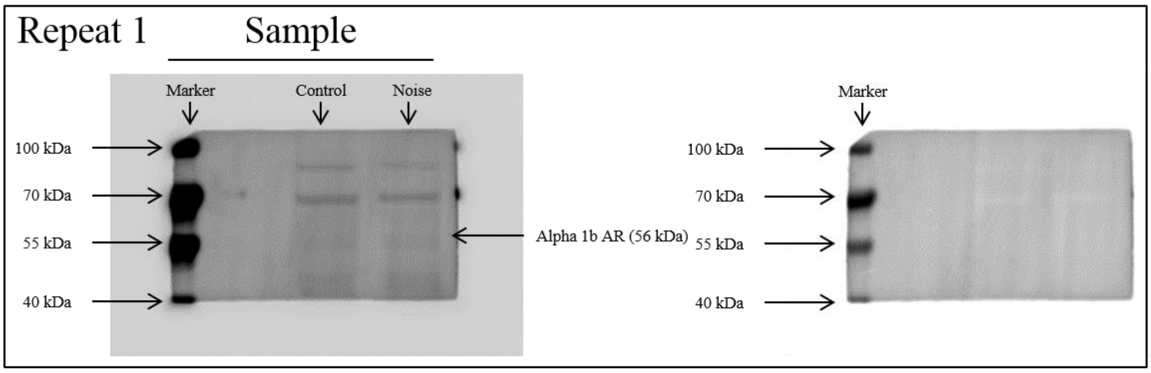

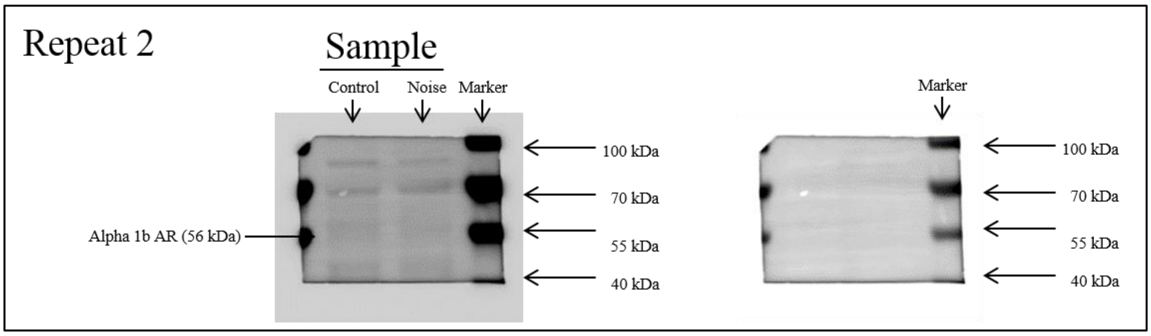

3. Alpha 1d AR (ab166925)

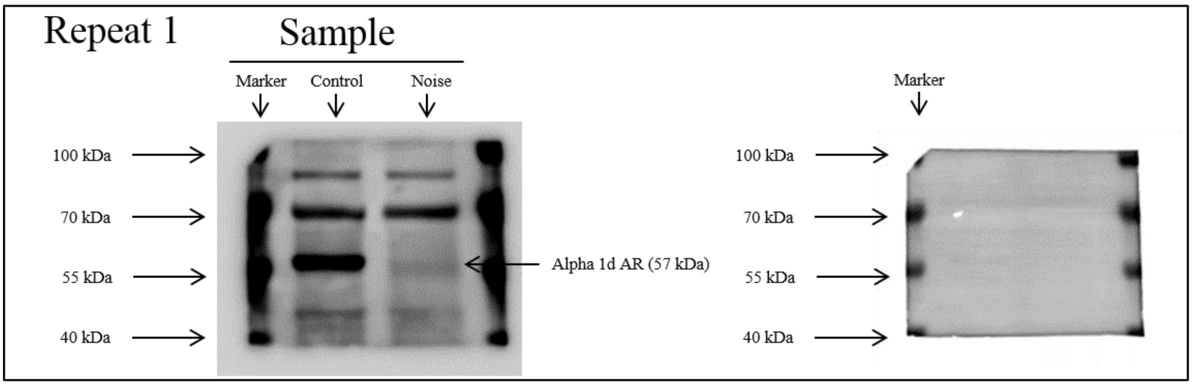

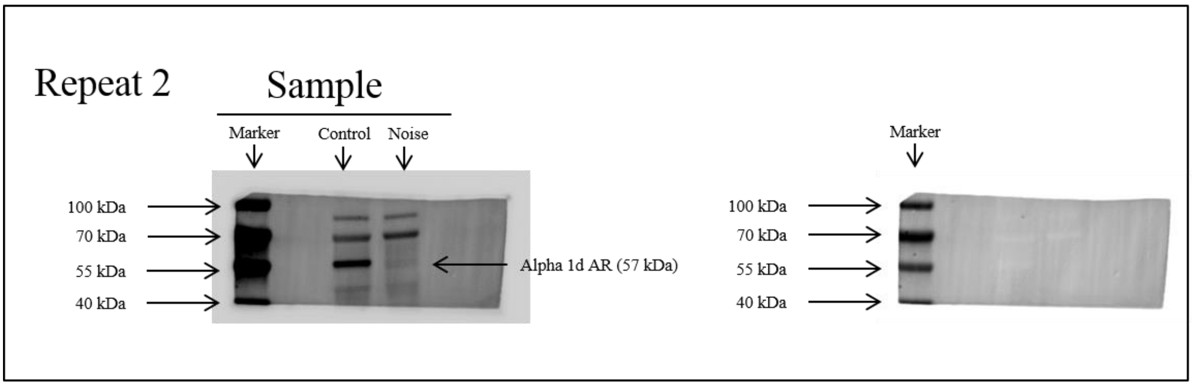

4. Alpha 2a AR (14266-1-AP)

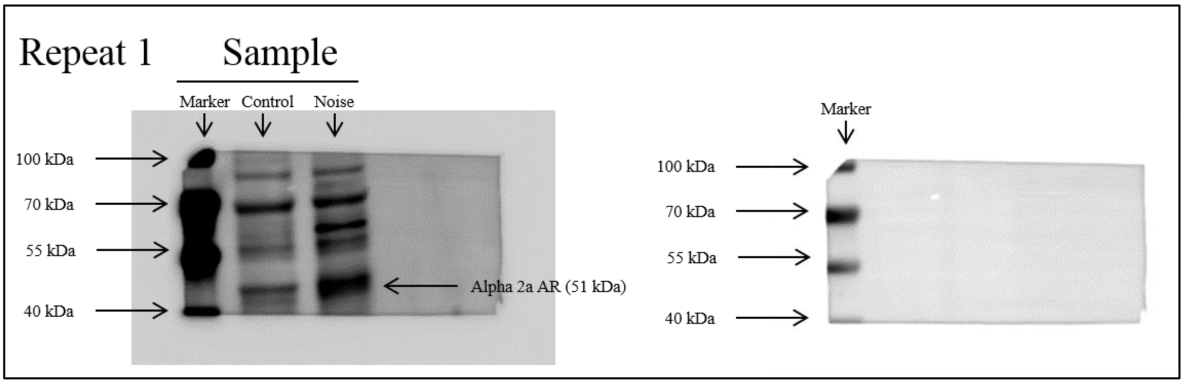

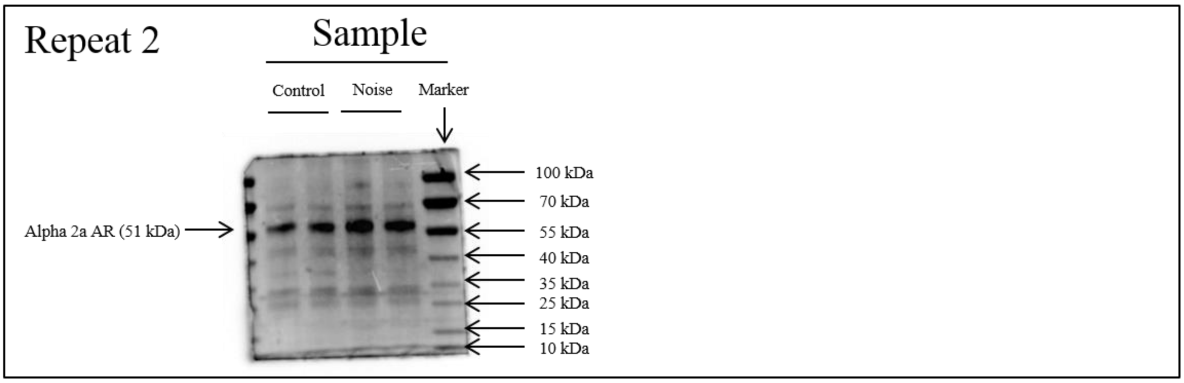

5. Alpha 2b AR (A8535)

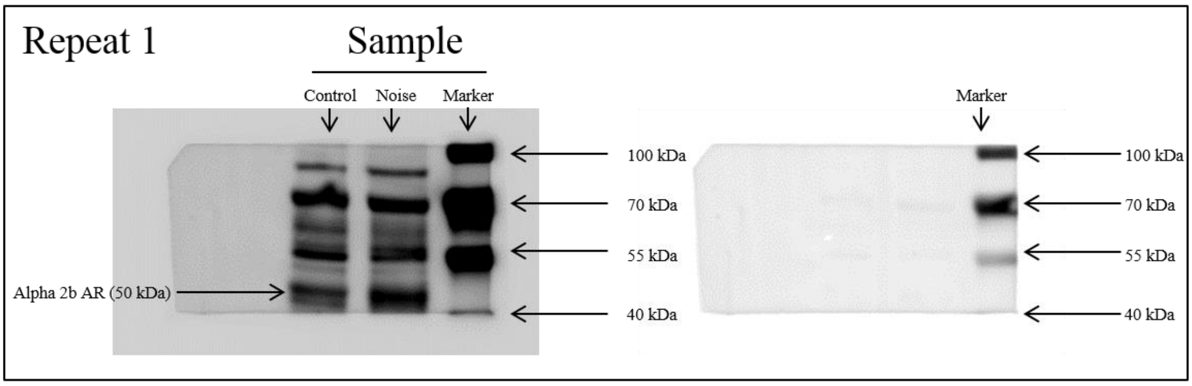

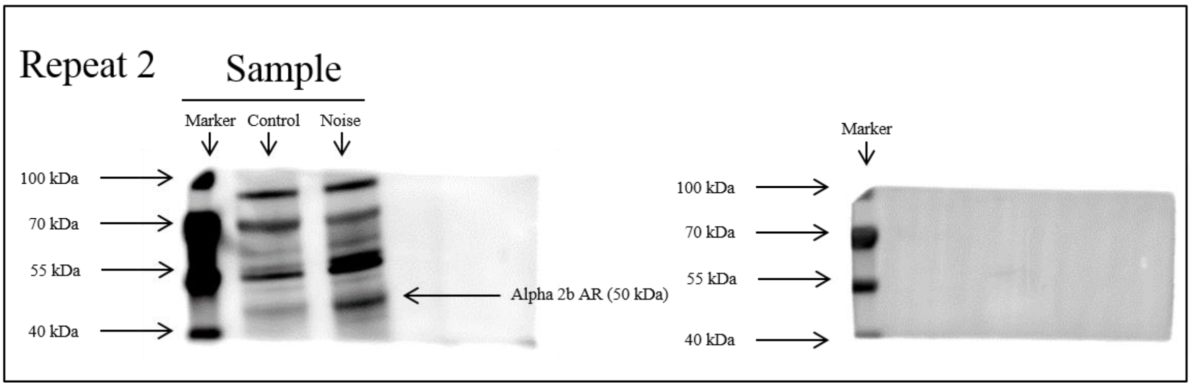

6. Alpha 2c AR (DF3108)

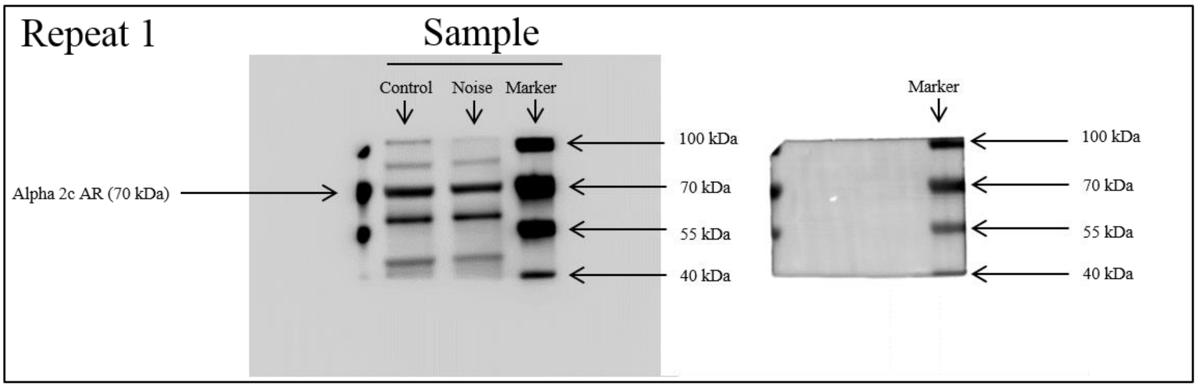

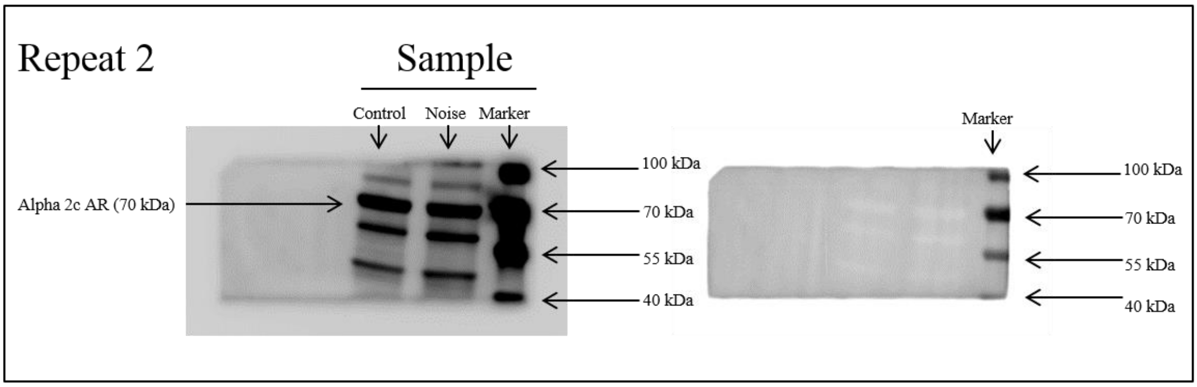

7. Beta 1 AR (bs-0498R)

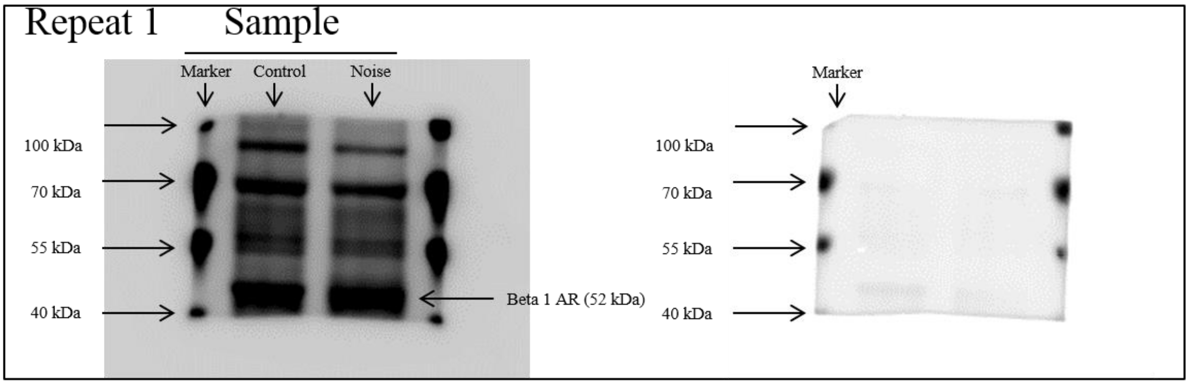

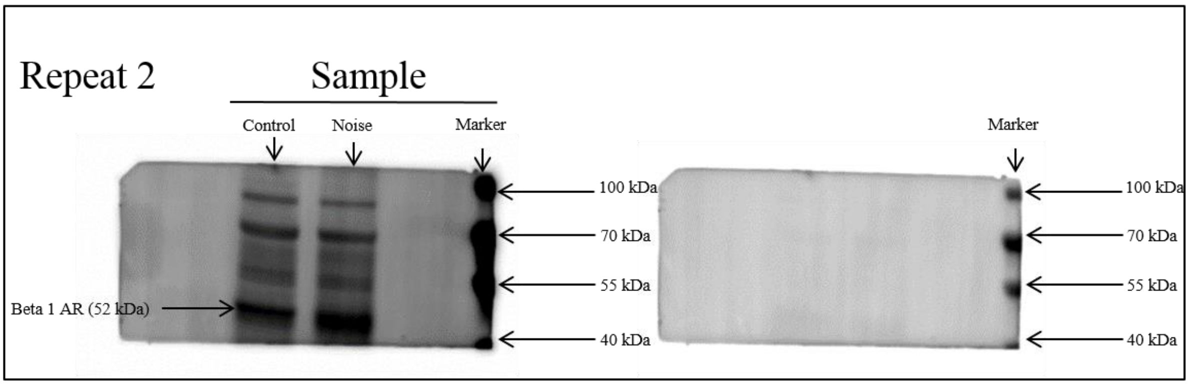

8. Beta 2 AR (DF3512)

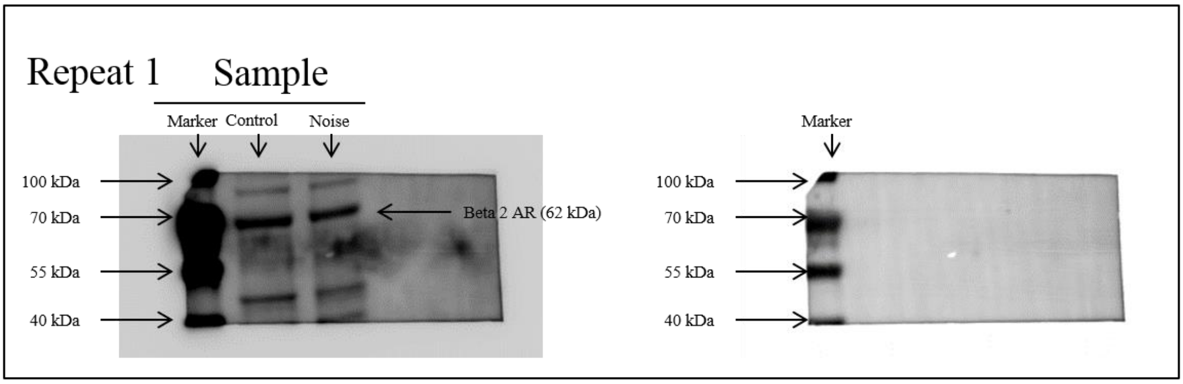

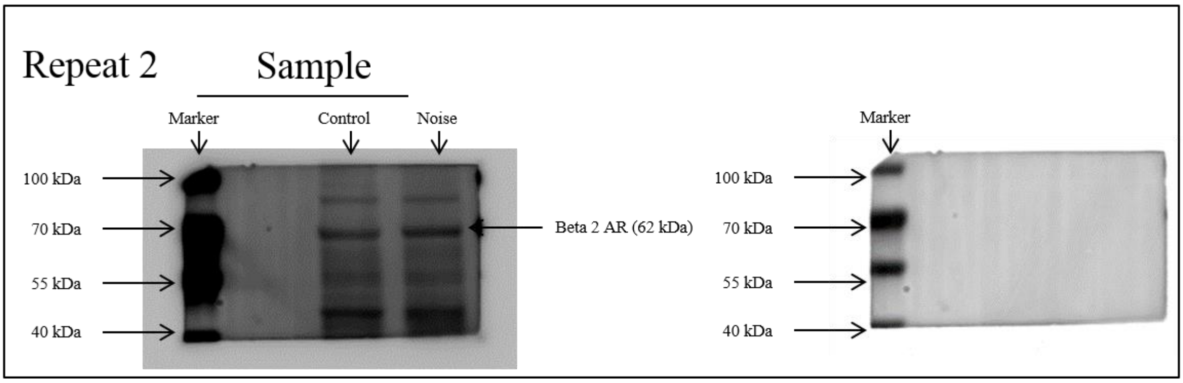

9. Beta 3 AR (bs-1063R)

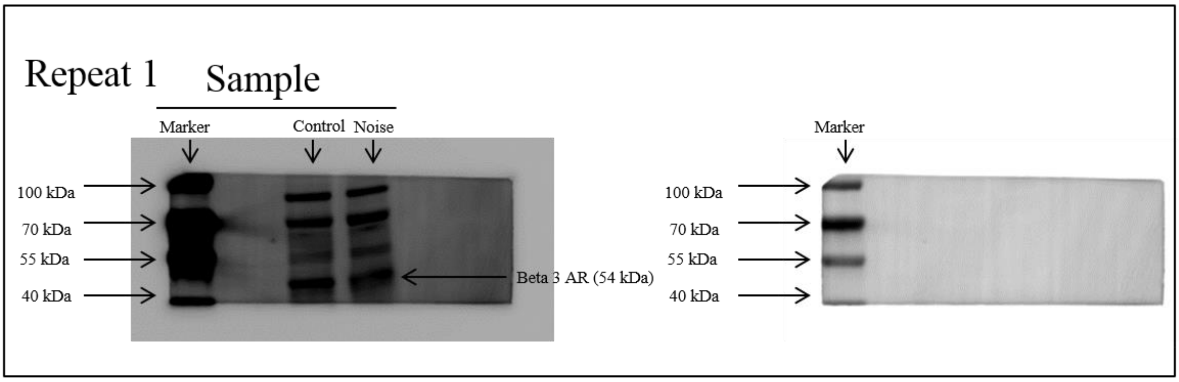

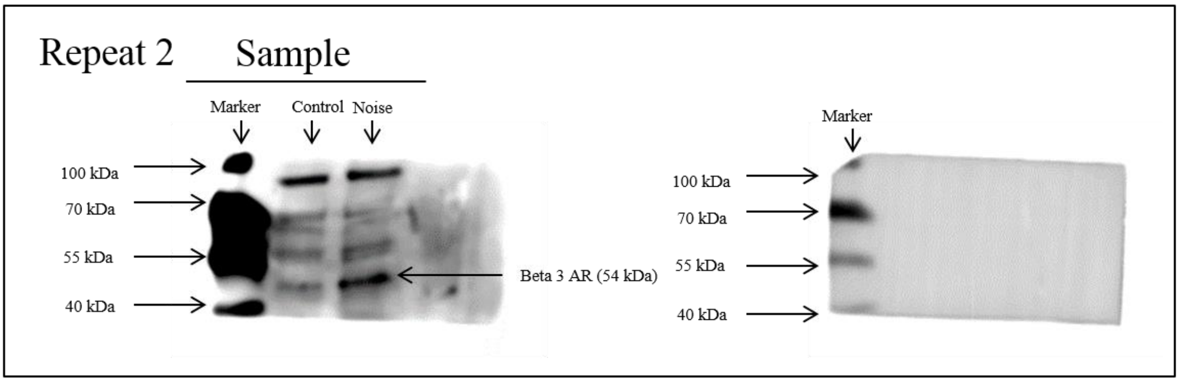

10. GAPDH (10494-1-AP)

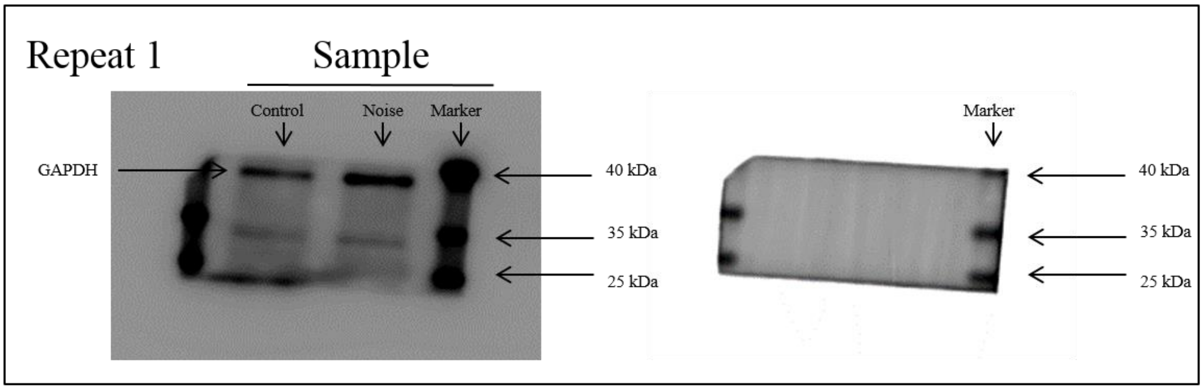

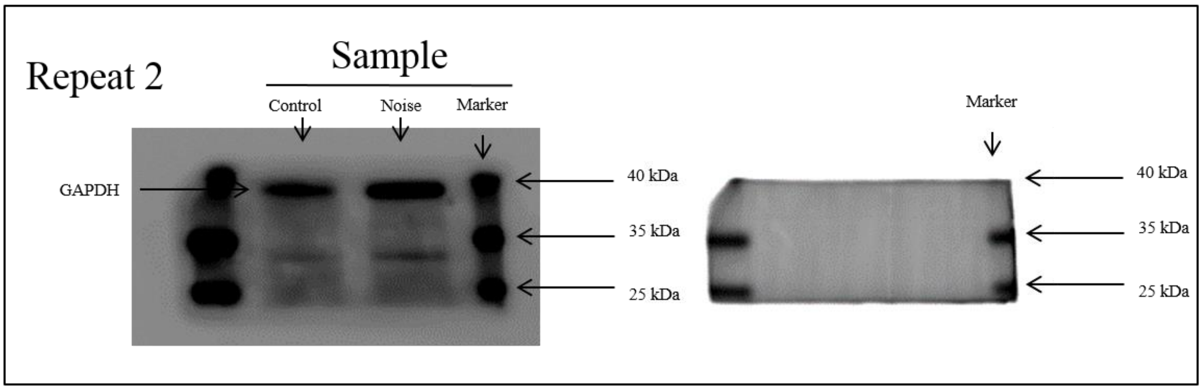

Supplement: Supplementary file 1 — Supplementary Material 1 [file 12868_2024_845_MOESM1_ESM.pdf]
